# Glutamate ionotropic receptor NMDA type subunit 2A (*GRIN2A*) gene polymorphism (rs4998386) and Parkinson's disease susceptibility: A meta‐analysis

**DOI:** 10.1002/agm2.12075

**Published:** 2019-07-23

**Authors:** Gaurav Nepal, Jessica Holly Rehrig, Rajeev Ojha

**Affiliations:** ^1^ Tribhuvan University Institute of Medicine Kathmandu Nepal; ^2^ University of New England College of Osteopathic Medicine Biddeford Maine USA; ^3^ Department of Neurology Tribhuvan University Institute of Medicine Kathmandu Nepal

**Keywords:** Excitotoxicity, glutamate ionotropic receptor NMDA type subunit 2A, Neurodegeneration, NMDA receptor, Parkinson's disease, rs4998386 polymorphism

## Abstract

**Objective:**

Dopaminergic neuronal degeneration seen in Parkinson's disease (PD) might result from a single nucleotide polymorphism (SNP) in the glutamate ionotropic receptor NMDA type subunit 2A (*GRIN2A*) gene. We thus performed a meta‐analysis exploring the relationship between the rs4998386 SNP of the *GRIN2A* gene and PD susceptibility.

**Methods:**

We searched PubMed, EMBASE, Web of Science, Google Scholar, and China National Knowledge Infrastructure for studies published between January 2005 and January 2019. The association between the rs4998386 polymorphism and PD susceptibility was evaluated by calculating the pooled odds ratios (ORs) and 95% confidence intervals (CIs).

**Results:**

Meta‐analysis results did not show a significant association between the rs4998386 polymorphism of the *GRIN2A* gene and PD susceptibility when assuming an allelic model (OR, 0.90; 95% CI, 0.76‐1.07; *P* = .22; *I* ^2 ^= 53%), a dominant model (OR, 0.96; 95% CI, 0.82‐1.12; *P* = .62; *I* ^2 ^= 64%), or a recessive model (OR, 1.14; 95% CI, 0.93‐1.38; *P* = .22; *I* ^2 ^= 0%).

**Conclusion:**

Our meta‐analysis found that the rs4998386 polymorphism of the *GRIN2A* gene is not associated with risk of PD in either Europeans or white Americans. However, large sample studies with different ethnicities should be conducted to establish the role of the rs4998386 polymorphism in PD pathophysiology.

## 1 INTRODUCTION

Parkinson's disease (PD) is the second most common neurodegenerative disease after Alzheimer's disease.^1^ PD is characterized by degeneration of dopaminergic neurons in the substantia nigra, which can present clinically as resting tremor, muscle rigidity, bradykinesia, and postural dysfunctions.^2^ Though James Parkinson's essay “Shaking Palsy” was published nearly 200 years ago, the exact pathophysiology of PD remains unknown. There are currently 6.1 million people with PD globally and the burden is increasing; thus, the creation of more therapeutic treatment options is urgently needed and will rely on further research.^3^

Current research hypothesizes PD as a compilation of complex interactions between genetic and environmental factors. PD was historically considered sporadic in nature, without genetic origin. In the past decade, however, genetic studies performed in various geographical regions worldwide have strengthened the hypothesis that PD has a substantial genetic component. Numerous genes have unequivocally been tied to familial PD, including *SNCA*, *PRKN*, *PINK1*,* DJ1*,* LRRK2*,* ATP13A2*, and *PLA2G6*.^4^, ^5^ This “familial form” of PD, however, accounts for only a small percentage of overall PD diagnoses. The majority of PD cases are considered a “sporadic form” of disease, indicating that additional factors may also contribute to the risk of PD.

Current evidence suggests an association between glutamate‐mediated excitatory neurotoxicity and neurodegenerative diseases.^6^, ^7^, ^8^ The excitatory effects of glutamate are exerted via activation of ionotropic receptors, mainly at NMDA receptors (NMDARs). NMDAR channels are heteromers composed of the key receptor subunit, NMDAR1, with one or more of the four NMDAR2 subunits: NMDAR2A, NMDAR2B, NMDAR2C, and NMDAR2D.^9^ Continuous activation of large numbers of NMDARs can lead to elevated intracellular calcium and catabolic enzyme activity, which triggers a cascade of events leading to cell death. These event cascades include mitochondrial membrane depolarization, caspases activation, cellular toxicity, and production of reactive oxygen species and nitrogen free radicals.^8^ A growing body of evidence supports an essential role for oxidative stress in the initiation and progression of neurodegeneration in PD.^10^, ^11^, ^12^

The substantia nigra pars compacta (SNPC), the location of the primary pathological lesion, is predisposed to oxidative stress as well as toxic and metabolic insults.^8^ One hypothesis suggests that mitochondria genetic alterations lead to impaired mitochondrial function and, therefore, degeneration of SNPC.^11^, ^12^ Oxidative stress may also be mediated by NMDAR‐induced production of reactive oxygen species, such as peroxide, hydroxyl radical, and superoxide. Animal studies have shown that both competitive and non‐competitive antagonism at the NMDAR lead to an anti‐parkinsonism effect.^13^ Currently, only one anti‐glutamatergic pharmacotherapy, amantadine, is used clinically to treat PD. Amantadine has been shown to noncompetitively block NMDARs at therapeutic concentrations in the central nervous system, supporting the association between NMDA and PD.^13^ Unfortunately, the precise role of NMDAR‐mediated excitotoxicity in PD progression has not been established and thus remains unclear.

With this supporting data, we hypothesized that dopaminergic neuronal degeneration seen in PD could result from a single nucleotide polymorphism (SNP) in the NMDAR. The glutamate ionotropic receptor NMDA type subunit 2A (*GRIN2A*) gene encodes for an epsilon subunit of NMDAR (ie, NMDAR2A). No studies have focused on an association between the SNP of the *GRIN2A* gene and PD susceptibility. However, four studies to date have established an interaction between rs4998386 polymorphisms of the *GRIN2A* gene and caffeine on PD risk. Though these studies were not performed to establish association between *GRIN2A* rs4998386 SNP and PD, we extracted relevant data from these studies to perform a meta‐analysis exploring the relationship between the rs4998386 SNP of the *GRIN2A* gene and PD susceptibility.

## 2 MATERIALS AND METHODS

This meta‐analysis was conducted in accordance with the guidance of the Preferred Reporting Items for Systematic Reviews and Meta‐Analyses (PRISMA) statement.^14^ All collected data were extracted from published studies, without ethical issues.

### 2.1 Literature search

We searched PubMed, EMBASE, Web of Science, Google Scholar, and China National Knowledge Infrastructure for studies published between January 2005 and January 2019. Searches were conducted using the following keywords and phrases: “*GRIN2A* gene polymorphism,” “glutamate ionotropic receptor NMDA type subunit 2A gene polymorphism,” “NMDAR2A gene polymorphism,” or “rs4998386 polymorphism” in combination with “Parkinson's disease,” “idiopathic disease,” or “neurodegenerative disease.” Titles and abstracts were screened and full text was acquired for all papers. Those that met inclusion criteria were manually reviewed for confirmation of their eligibility. Screening, report retrieval, and inclusion/exclusion of studies were conducted by two authors (G. N. and R. O.). Any uncertainty about study eligibility was resolved through discussion with the third author.

### 2.2 Eligibility criteria

Studies conducted on human subjects were included, without limitation of study language. In this meta‐analysis, all included studies met the following criteria: (a) studies focused on the association between rs4998386 polymorphism and PD susceptibility, (b) patients with PD were diagnosed using standard diagnostic criteria by a neurologist, and (c) there were sufficient data of the genotypes in the case‐control groups to evaluate the odds ratios (ORs) and 95% confidence intervals (CIs). The exclusion criteria were: (a) publications with overlapping cases and controls from a similar study, (b) no genotypic data available, and (c) publications with patients with other forms of parkinsonism.

### 2.3 Data abstraction and assessment of methodological quality

Relevant data from each study, along with their supplementary files, were independently extracted by two reviewers (G. N. and R. O.) using a standardized, structured methodology that included the following: first author, type of design, site of study, patient ethnicity, year of publication, sample size of cases and controls, control design, Hardy‐Weinberg equilibrium, source of DNA, genotyping method, and data of the genotypes in the case‐control groups. The methodological quality of each study was assessed independently by two reviewers (G. N. and R. O.) using the parameters previously used by Kumar et al.^15^ In this scale, the following sections were included for assessment of methodological quality: representativeness of cases, source of controls, matching of controls, ascertainment of cases, ascertainment of controls, blinding while genotyping, genotyping methods, Hardy‐Weinberg equilibrium, and association assessment. A maximum score of 2 can be provided for each section listed, except source of controls (maximum 3), ascertainment of controls (maximum 1), blinding while genotyping (maximum 1), and association assessment (maximum 1). Methodological quality of scale score varies from 0 to 16, in which higher scores represent better quality and lower scores represent lower quality. Any discrepancies during data extraction and quality assessment were resolved by discussion with the third author.

### 2.4 Statistical analysis

The association between the rs4998386 polymorphism and PD susceptibility was evaluated by calculating the pooled ORs and 95% CIs. Heterogeneity between the included studies was determined with Cochran's *Q* test and the *I* ^2^ test. The presence of *P* values above .1 or *I* ^2^ more than 50% was considered an indicator of significant heterogeneity. When significant heterogeneity was present, we selected the random‐effects model to calculate the effects size. Three genetic models were examined: the allelic model (T allele vs C allele), the dominant model (TT + TC vs CC), and the recessive model (TT vs CC + TC). Subgroup analyses were conducted according to geographic location. Sensitivity analysis was performed to examine the stability of analysis. Egger's linear regression test and Begg's test were used to identify publication biases. Statistical analysis was performed using Comprehensive Meta‐Analysis software (CMA 3.3, Biostat, 2014).

## 3 RESULTS

### 3.1 Literature search

**Figure 1:**
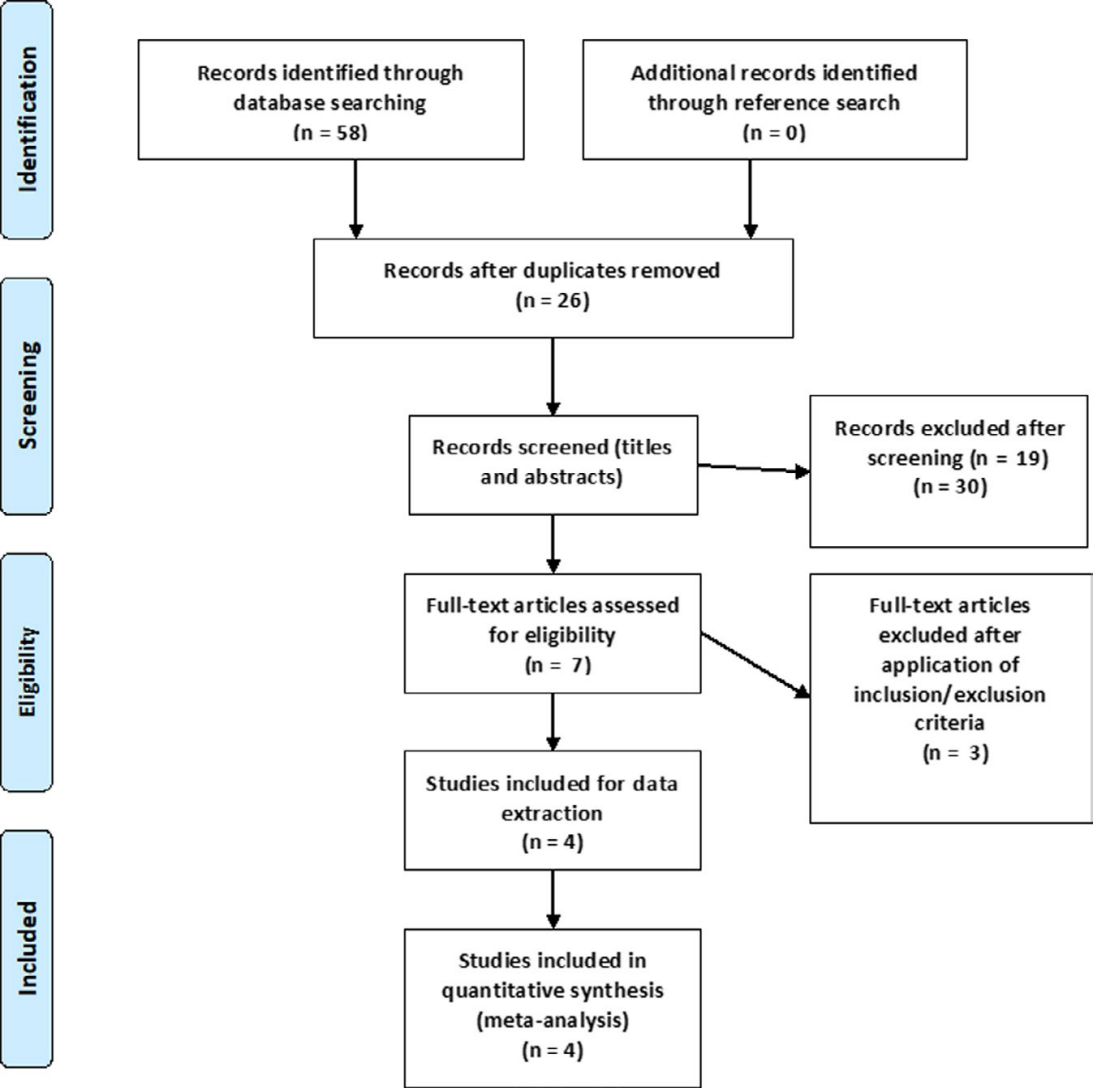
Flow of systematic literature search and selection

The results of the systematic literature search and selection are summarized in Figure 1. We identified 58 articles through various database searching. After excluding duplicates, 26 articles remained. Screening titles and abstracts of these 26 articles resulted in seven relevant abstracts. After inclusion and exclusion criteria, four studies were chosen for final data analysis.

### 3.2 Study and patient characteristics

Our research included four studies with data from 12 different cohorts. All studies used a case‐control design. There were 5783 cases and 8788 controls in our study. Hamza et al used four different cohorts from the United States in their study: (i) NeuroGenetics Research Consortium (NGRC); (ii) Parkinson, Environment and Gene (PEG); (iii) Parkinson's, Genes, and Environment (PAGE) from the prospective NIH‐AARP Diet and Health Study cohort; and (iv) Hussman Institute for Human Genomics (HIHG).^16^ Yamada‐Fowler et al used a Swedish cohort.^17^ Ahmed et al used two cohorts from the United States, and one from France and Denmark, respectively.^18^ The study by Kim et al was composed of participants from three longitudinal cohorts: the Nurses’ Health Study (NHS), the Health Professionals Follow‐up Study (HPFS), and the Cancer Prevention Study II Nutrition Survey cohort (CPS‐IIN).^19^ Allelic and genotypic data from each cohort are illustrated in Table 1.

**Table 1:** Key methodological characteristics of included studies

Author	Year of publication	Design	Study cohort	Country	Ethnicity	Sample size (case/control)	Genotypes distribution (case/control)			Allelic distribution (case/control)	
							CC	TC	TT	T	C
Hamza et al^16^	2011	Case‐control	NGRC	United States	Caucasian	1458/931	1227/716	219/204	12/11	216/200	2700/1662
			PEG	United States	Caucasian	280/310	234/249	42/55	4/6	46/60	514/560
			PAGE	United States	Caucasian	525/1474	434/1199	85/267	6/8	88/258	962/2690
			HIHG	United States	Caucasian	209/133	169/110	36/22	4/1	39/22	379/244
Yamada‐Fowler et al^17^	2014	Case‐control	Swedish population	Sweden	Caucasian	193/377	159/284	30/88	4/5	38/98	348/656
Ahmed et al^18^	2014	Case‐control	French population	France	Caucasian	300/598	242/492	56/101	2/5	NA	NA
			Danish population	Denmark	Caucasian	1288/1394	1042/1094	232/286	14/14	NA	NA
			Seattle population	United States	Caucasian	386/502	302/418	77/81	7/3	NA	NA
			Rochester population	United States	Caucasian	315/315	247/244	68/68	0/3	NA	NA
Kim et al^19^	2018	Case‐control	HPFS	United States	Caucasian	286/964	242/809	43/146	1/9	45/164	527/1764
			NHS	United States	Caucasian	393/1625	301/1291	90/320	2/14	94/348	692/2902
			CPS‐IIN	United States	Caucasian	150/165	114/130	36/35	0/0	36/35	264/295

Abbreviations: CPS‐IIN, Cancer Prevention Study II Nutrition Survey; HIHG, Hussman Institute for Human Genomics; HPFS, Health Professionals Follow‐up Study; NA, Not available; NGRC, NeuroGenetics Research Consortium; NHS, Nurses’ Health Study; PAGE, Parkinson's, Genes, and Environment from the prospective NIH‐AARP Diet and Health Study; PEG, Parkinson, Environment and Gene.

Of the 12 different cohorts included in our study, nine were from the United States, with all included participants of Caucasian race. Methods of PD case and control ascertainment are given in Table 2. All included cohorts reached Hardy‐Weinberg equilibrium in their control population. For genotyping, the TaqMan assay method was most commonly used among cohorts, though some applied the Illumina HumanOmni1‐Quad v1.0, Sequenom MassARRAY and Perlegen platform. All cohorts used population‐based controls with DNA sourced from whole blood, saliva, and buccal samples. According to quality‐assessment tools, the methodological quality score ranged from 8 to 12, meaning no study analyzed was of low quality. Detailed study and patient characteristics are tabulated in Table 1.

**Table 2:** Characteristics of subjects included in each study

Author	Study cohort	PD case ascertainment	Control selection	DNA source	Genotyping method	Hardy‐Weinberg equilibrium
Hamza et al^16^	NGRC	Clinic‐based sequential ascertainment of PD cases	Matched for age, sex, race	Whole blood	Illumina HumanOmni1‐Quad v1.0	Confirmed
	PEG	Community‐based incident cases of PD	Matched for age, sex, race	Whole blood (all PD cases and half of controls) or saliva (half of controls)	TaqMan assay	Confirmed
	PAGE	Community‐based incident cases of PD	Matched for age, sex, race	Saliva	Sequenom MassARRAY	Confirmed
	HIHG	Clinic‐based and self‐referral cases of PD	Matched for age, sex, race	Whole blood	TaqMan assay	Confirmed
Yamada‐Fowler et al^17^	Swedish population	Single clinic‐based l‐dopa‐positive PD patients	Matched for age, sex	Whole blood	TaqMan assay	Confirmed
Ahmed et al^18^	French population	PD cases identified from health insurance system	Randomly drawn from the health insurance system and matched for age, sex, district of residency	Saliva	TaqMan assay	Confirmed
	Danish population	PD patients identified from National Hospital Register files	Randomly drawn from Danish Central Population Registry and matched for age, sex	Saliva	TaqMan assay	Confirmed
	Seattle population	Newly diagnosed PD cases were identified in a population‐based case‐control study conducted in Seattle	Controls had no neurological disorder and matched for sex, age, race, ethnicity	Whole blood (86%) or buccal specimens (14%)	TaqMan assay	Confirmed
	Rochester population	PD cases recruited from family‐based dataset consisting of discordant sibling pairs, such that each sibling pair has one member affected with PD and one unaffected	Controls were siblings without parkinsonism and matched for age and sex	Whole blood	Perlegen platform	Confirmed
Kim et al^19^	HPFS	PD cases confirmed by neurologist from HPFS cohort	Randomly drawn from same cohort and matched for sex, age, race, source of DNA	Buccal sample	TaqMan assay	Confirmed
	NHS	PD cases confirmed by neurologist from NHS cohort	Randomly drawn from same cohort and matched for sex, age, race, source of DNA	Buccal sample	TaqMan assay	Confirmed
	CPS‐IIN	PD cases confirmed by neurologist from CPS‐IIN cohort	Randomly drawn from same cohort and matched for sex, age, race, source of DNA	Buccal sample	TaqMan assay	Confirmed

Abbreviations: CPS‐IIN, Cancer Prevention Study II Nutrition Survey; HIHG, Hussman Institute for Human Genomics; HPFS, Health Professionals Follow‐up Study; NGRC, NeuroGenetics Research Consortium; NHS, Nurses’ Health Study; PAGE, Parkinson's, Genes, and Environment from the prospective NIH‐AARP Diet and Health Study; PD, Parkinson's disease; PEG, Parkinson, Environment and Gene.

### 3.3 Meta‐analysis

**Figure 2:**
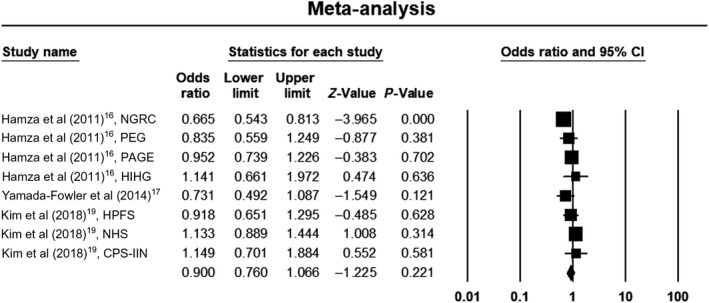
Forest plot of the results for allelic model. CI, confidence interval; CPS‐IIN, Cancer Prevention Study II Nutrition Survey; HIHG, Hussman Institute for Human Genomics; HPFS, Health Professionals Follow‐up Study; NGRC, NeuroGenetics Research Consortium; NHS, Nurses’ Health Study; PAGE, Parkinson's, Genes, and Environment from the prospective NIH‐AARP Diet and Health Study; PEG, Parkinson, Environment and Gene

**Figure 3:**
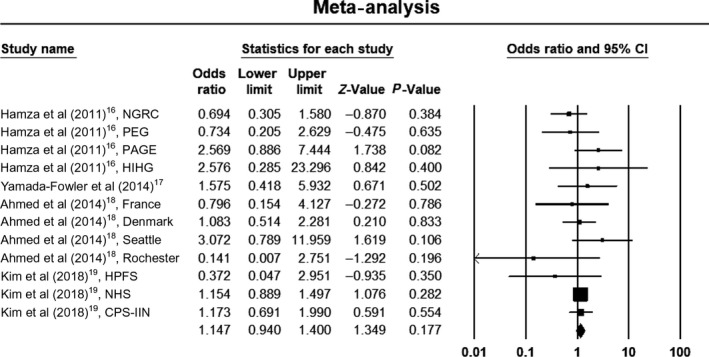
Forest plot of the results for recessive model. CI, confidence interval; CPS‐IIN, Cancer Prevention Study II Nutrition Survey; HIHG, Hussman Institute for Human Genomics; HPFS, Health Professionals Follow‐up Study; NGRC, NeuroGenetics Research Consortium; NHS, Nurses’ Health Study; PAGE, Parkinson's, Genes, and Environment from the prospective NIH‐AARP Diet and Health Study; PEG, Parkinson, Environment and Gene

**Figure 4:**
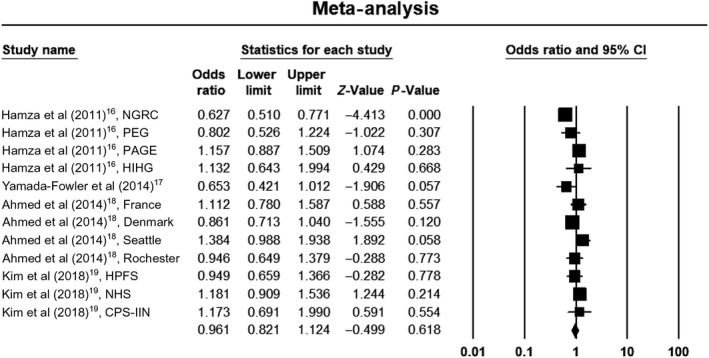
Forest plot of the results for dominant model. CI, confidence interval; CPS‐IIN, Cancer Prevention Study II Nutrition Survey; HIHG, Hussman Institute for Human Genomics; HPFS, Health Professionals Follow‐up Study; NGRC, NeuroGenetics Research Consortium; NHS, Nurses’ Health Study; PAGE, Parkinson's, Genes, and Environment from the prospective NIH‐AARP Diet and Health Study; PEG, Parkinson, Environment and Gene

Due to significant heterogeneity within studies in the allelic and dominant models, we used a random‐effects model to estimate pooled ORs and 95% CIs. The recessive model was without heterogeneity, so we used a fixed‐effects model to estimate pooled ORs and 95% CIs. Meta‐analysis results did not show a significant association between the rs4998386 polymorphism of the *GRIN2A* gene and PD susceptibility when assuming an allelic model (OR, 0.90; 95% CI, 0.76‐1.07; *P* = .22; *I* ^2 ^= 53%), a dominant model (OR, 0.96; 95% CI, 0.82‐1.12; *P* = .62; *I* ^2 ^= 64%), or a recessive model (OR, 1.14; 95% CI, 0.93‐1.38; *P* = .22; *I* ^2 ^= 0%). Forest plots of the results for the allelic, recessive, and dominant models are demonstrated in Figures 2, 3, 4, respectively.

**Figure 5:**
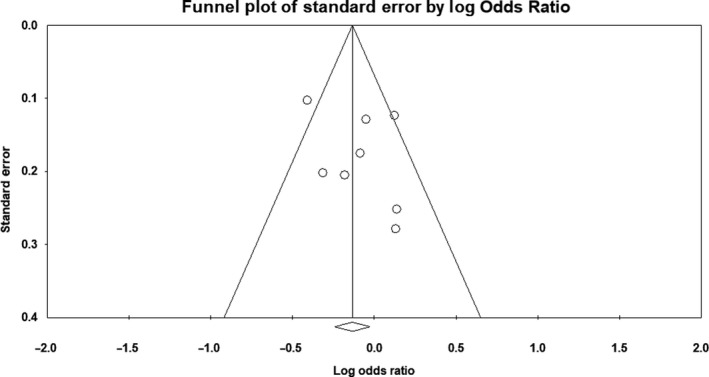
Funnel plot for detection of publication bias in allelic model

**Figure 6:**
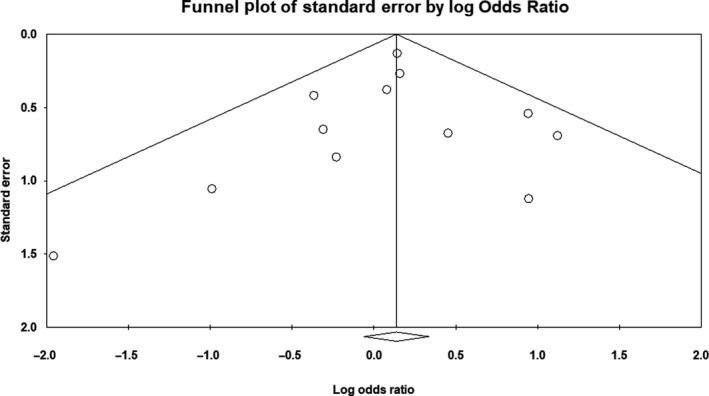
Funnel plot for detection of publication bias in recessive model

**Figure 7:**
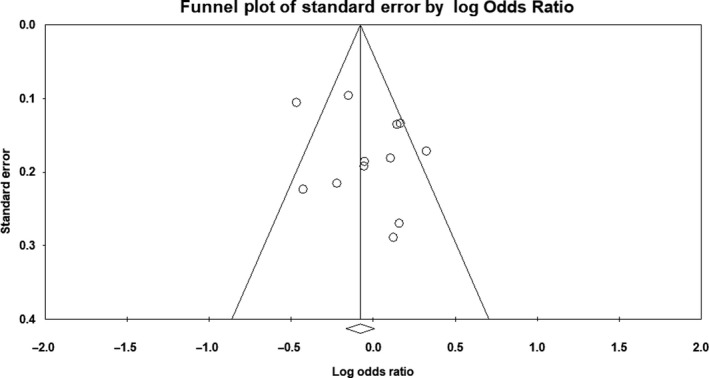
Funnel plot for detection of publication bias in dominant model

There was no evidence of publication bias on the allelic model (Egger's test: *P* = .36; Begg's test: *P* = .71), recessive model (Egger's test: *P* = .75; Begg's test: *P* = .53), or dominant model (Egger's test: *P* = .28; Begg's test: *P* = .63). In addition, on inspection of the funnel plot, publication bias was not obvious. Funnel plots for the detection of publication bias in the allelic, recessive, and dominant models are demonstrated in Figures 5, 6, 7, respectively.

For all three genetic models, we sequentially excluded a single study from the pooled analysis and recalculated the summary ORs to check whether the summary ORs were significantly changed. The recalculated ORs also indicated stability of analysis. We performed a subgroup analysis based on the geographical location of the study (Europe vs America). In the allelic model, the rs4998386 polymorphism was not associated with PD in either Europeans (OR, 0.73; 95% CI, 0.49‐1.09; *P* = .12) or white Americans (OR, 0.92; 95% CI, 0.77‐1.11; *P* = .4). In the dominant model, the rs4998386 polymorphism was not associated with PD in either Europeans (OR, 0.87; 95% CI, 0.69‐1.11; *P* = .26) or white Americans (OR, 1; 95% CI, 0.81‐1.23; *P* = 1). Similarly, in the recessive model, the rs4998386 polymorphism was not associated with PD in either Europeans (OR, 1.12; 95% CI, 0.61‐2.04; *P* = .72) or white Americans (OR, 1.13; 95% CI, 0.92‐1.40; *P* = .24).

## 4 DISCUSSION

While the NMDAR is involved in synaptic plasticity and learning, its excessive activation results in excitotoxic neuronal damage, which is hypothesized to be a mechanism underlying several neurologic and neurodegenerative disorders. Increased glutamatergic transmission through the NMDAR is believed to play a role in the degeneration of the nigrostriatal dopaminergic pathway seen in PD pathophysiology. This deterioration leads to significant changes in striatal circuit structure and function, including modifications of the corticostriatal glutamatergic synaptic architecture and, consequently, loss of striatal synaptic plasticity and dopaminergic neurons.^20^ In addition, continuous mass activation of NMDARs increases intracellular calcium loads and catabolic enzyme activities, triggering a cascade that leads to cell death.^8^ Various animal models of PD have shown that NMDAR antagonists alleviate symptoms of PD.^13^ In animal models, NMDAR antagonists are effective antiparkinsonian agents and can reduce the complications of chronic dopaminergic therapy, such as “wearing off” and dyskinesias.^21^ Amantadine, an NMDAR antagonist, has been used clinically for treatment of dyskinesias and motor fluctuations in PD.^13^

The neuronal NMDAR signaling has been found to be an important pathway in the development and progression of schizophrenia,^22^, ^23^ epilepsy,^24^, ^25^ Huntington's disease,^26^, ^27^, ^28^ depression,^29^ Alzheimer's disease,^30^ and other neurodevelopmental disorders.^25^ Recently, some studies have explored the interaction between genetic alterations of NMDAR, coffee intake, and risk of PD. Hamza et al and Yamada‐Fowler et al found *GRIN2A* to be a PD modifier gene via interaction with coffee.^16^, ^17^ However, results from studies of Ahmed et al and Kim et al were not in favor of an interaction between caffeinated coffee consumption and rs4998386 for PD risk.^18^, ^19^

Apart from *GRIN2A*, genetic alteration in other subunits of NMDARs are also associated with PD. A study by Wu et al found that polymorphisms in the *GRIN1* and *GRIN2B* genes may serve as potential biomarkers for a reduced risk of PD among the Chinese population in Taiwan.^31^ Lee et al and Abidin et al found that polymorphisms in the *GRIN2B* gene were associated with an increased risk of developing impulse‐control behaviors among PD patients.^32^, ^33^ Therefore, genetic alteration in subunits of NMDARs do have involvement in the pathophysiology of PD. No study has primarily studied the association between the rs4998386 polymorphism of the *GRIN2A* gene of NMDAR and PD risk. Therefore, we conducted a meta‐analysis to examine the association between the rs4998386 polymorphism of the *GRIN2A* gene and PD risk.

Our meta‐analysis found that the rs4998386 polymorphism is not associated with PD in Europeans or white Americans in any of the three genetic models, though our results may have been influenced by several factors. Given assumptions that PD is multi‐determined and that the *GRIN2A* polymorphism alone is uncommon and small in effect, our power to detect genetic effects may have been reduced. PD is a multigenic disorder, with combined effects of multiple genotype polymorphisms predisposing carriers to increased PD risk. In two of the three genetic models, statistically significant heterogeneity was evident. Several aspects may contribute to this heterogeneity, including the diagnostic criteria used, early/late‐onset PD ratio, sex ratio, mean allele frequency, methods of genotyping, and source of DNA. Moreover, heterogeneity may also be due to the variations in age and environmental factors.

In addition, the following study limitations should be acknowledged. First, only people of European ancestry were included in our study. Therefore, results should be cautiously interpreted. Second, some useful information, such as diagnostic criteria, clinical stage of PD, onset age of PD patients, and other environmental factors, was not available. Third, significant heterogeneity was evident in our meta‐analysis. Lastly, gene‐gene interaction and gene‐environment interaction could not be analyzed due to lack of sufficient data.

## 5 CONCLUSION

Our meta‐analysis found that the rs4998386 polymorphism of the *GRIN2A* gene is not associated with risk of PD in either Europeans or white Americans. However, considering the study limitations, large sample studies with different ethnicities should be conducted to establish the role of the rs4998386 polymorphism in PD pathophysiology. Furthermore, age of onset, sex, and environmental factors that interact with rs4998386 polymorphisms should be investigated.

## AUTHOR CONTRIBUTIONS

G. N. and J. R. designed the study. G. N. and R. O. carried out the literature search, review, and selection. G. N. and J. R. carried out the statistical analysis and drafted the manuscript. All authors were involved in revising the manuscript critically for important intellectual content. All authors read and approved the final manuscript.

## CONFLICTS OF INTEREST

The authors declare no conflicts of interest.
